# Tea consumption and the risk of ovarian cancer: A meta-analysis of epidemiological studies

**DOI:** 10.18632/oncotarget.16890

**Published:** 2017-04-06

**Authors:** Xin Zhan, Jie Wang, Shufen Pan, Caijuan Lu

**Affiliations:** ^1^ Obstetrics and Gynecology Department, Hangzhou First People's Hospital, Hangzhou, Zhejiang 310006, PR China; ^2^ Obstetrics and Gynecology Department, The First Affiliated Hospital of Zhejiang Chinese Medical University Zhejiang Provincial Hospital of TCM, Hangzhou, Zhejiang 310016, PR China; ^3^ Department of Obstetrics and Gynecology, Central Hospital of Wenzhou, Luchengqu, Wenzhou, Zhejiang 325000, PR China

**Keywords:** ovarian cancer, tea, meta-analysis

## Abstract

A large number of epidemiological studies have provided conflicting results about the relationship between tea consumption and ovarian cancer. This study aimed to clarify the association between tea consumption and ovarian cancer. A literature search of the MEDICINE, Scopus, PubMed, and Web of Science databases was performed in April 2016. A total of 18 (11 case-control and 7 cohort) studies, representing data for 701,857 female subjects including 8,683 ovarian cancer cases, were included in the meta-analysis. A random-effects meta-analysis was used to compute the pooled relative risks (RR), meta regression, and publication bias, and heterogeneity analyses were performed for the included trials. We found that tea consumption had a significant protective effect against ovarian cancer (relative risk [RR] = 0.86; 95% confidence interval [CI]: 0.76, 0.96). The relationship was confirmed particularly after adjusting for family history of cancer (RR = 0.85; 95% CI: 0.72, 0.97), menopause status (RR = 0.85; 95% CI: 0.72, 0.98), education (RR = 0.82; 95% CI: 0.68, 0.96), BMI (RR = 0.85; 95% CI: 0.70, 1.00), smoking (RR = 0.83; 95% CI: 0.72, 0.93) and Jadad score of 3 (RR = 0.76; 95% CI: 0.56, 0.95) and 5 (RR = 0.74; 95% CI: 0.59, 0.89). The Begg's and Egger's tests (all *P* > 0.01) showed no evidence of publication bias. In conclusion, our meta-analysis showed an inverse association between tea consumption and ovarian cancer risk. High quality cohort-clinical trials should be conducted on different tea types and their relationship with ovarian cancer.

## INTRODUCTION

Ovarian cancer has the sixth-highest prevalence among cancers worldwide and is the primary cause of death due to gynecologic malignancy [1, 2]. Because most patients with cancer have been diagnosed at an advanced stage, its mortality rate is high and < 50% of patients live beyond 5 years after diagnosis [3]. The incidence of ovarian cancer widely differs among regions, races, and ethnicities: The incidence in the Asian population is much lower than that in the European population, indicating that lifestyle and eating habits may play an important role in the pathogenesis of ovarian cancer [4]. The influence of lifestyle on cancer has been reported in many studies. Changes in lifestyle factors, including diet, can prevent several types of cancers such as colorectal cancer, bladder cancer, ovarian cancer, and liver cancer [5–7]. Many *in vitro* and animal experiments have shown that tea contains a variety of complexes, especially polyphenols (green tea), which play a significant role in inhibiting the growth of cancer cells [8, 9].

Several epidemiological studies including case-control and cohort studies have investigated the association between tea consumption and ovarian cancer risk; however, their results were inconsistent. In 2015, Zhang et al. [10] performed a meta-analysis of observational studies that investigated the association between green tea intake and ovarian cancer risk and reported that high tea consumption had no significant effect on the risk of many cancers, including gastric, rectal, lung, colon, pancreatic, liver, breast, ovarian, prostate, and bladder cancers. However, their meta-analysis only included 6 observational studies, and their methodology was not comprehensive, as it did not include sub-group analyses according to the geographic location, adjustment for factors, Jadad scores from the literature, sensitivity analysis, and meta-regression analysis. Therefore, they were unable to identify potential sources of heterogeneity. In addition, no statistical significance was reported between tea consumption and ovarian cancer risk in 2 other meta-analyses [11, 12]. However, in another meta-analysis, tea consumption was found to be inversely, but not significantly, associated with ovarian cancer risk [13].

In order to clarify whether tea consumption is associated with ovarian cancer risk, this study aimed to perform a comprehensive meta-analysis of 18 epidemiological studies.

## RESULTS

### Literature search and study characteristics

**Figure 1:**
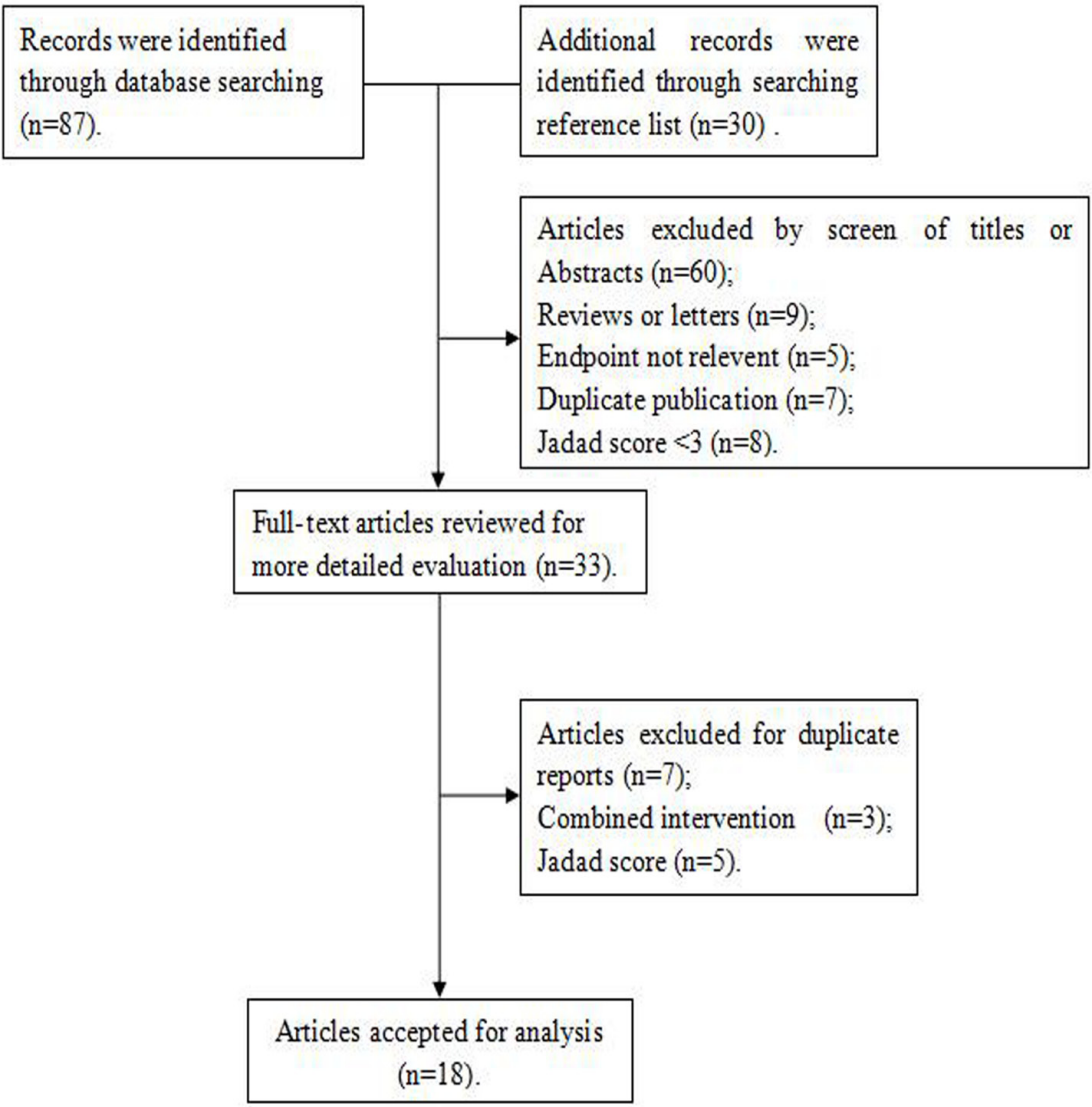
Search strategy and selection of studies

**Figure 2:**
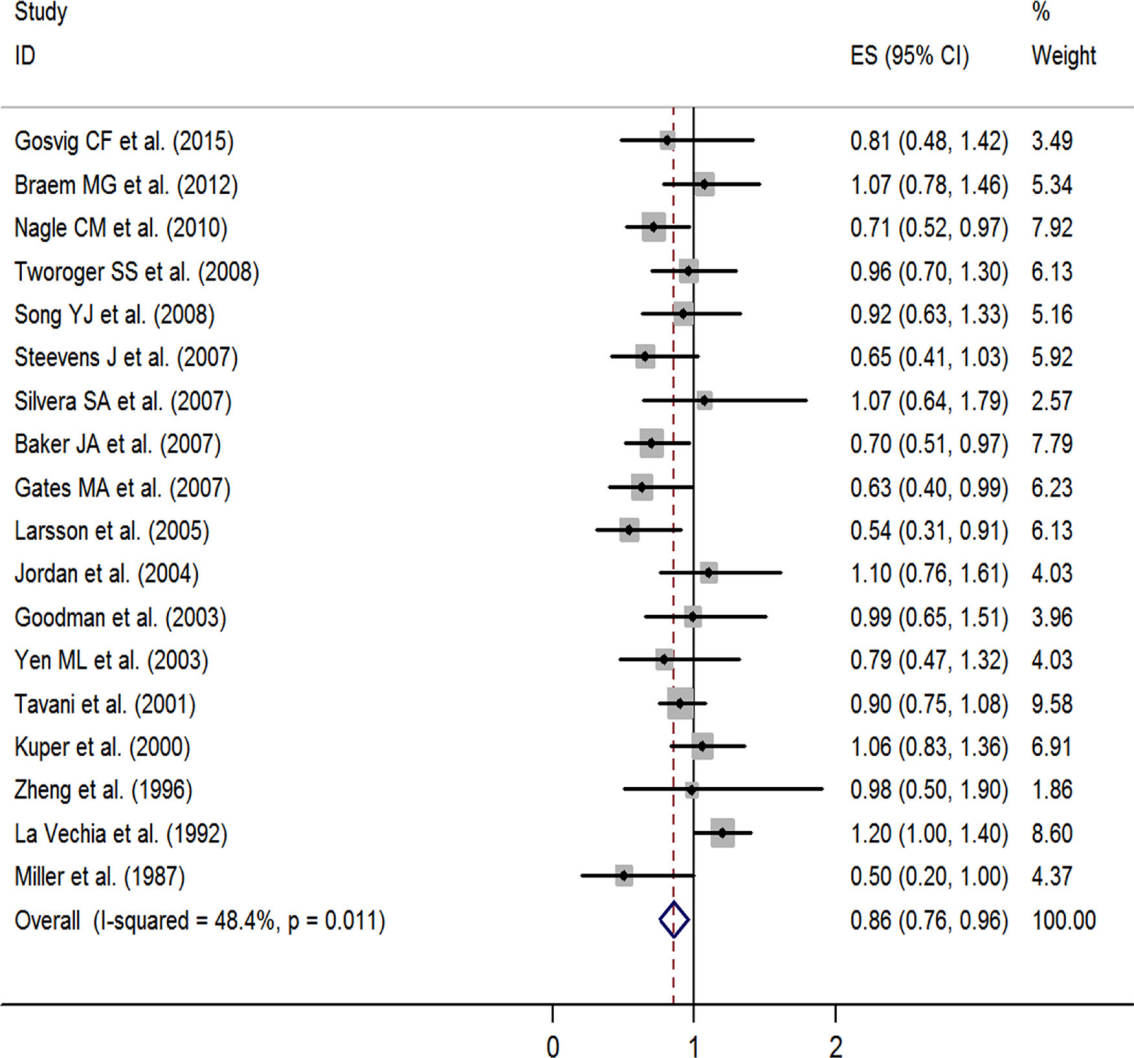
Forest plot of studies evaluating the association between tea consumption and risk of ovarian cancer, ES: effect size

Figure 1 illustrates the search process and the final selection of relevant studies. A total of 87 records were identified through database searching, and 30 additional records were identified through examination of reference lists. On the basis of the titles and abstracts, we identified 33 full-text articles. After further evaluation, 15 studies were excluded due to the lack of available data, duplicated reports, and Jadad score < 3. Finally, 18 [12–29] eligible studies published between 1987 and 2015 were identified, including 11 case-control studies [14, 15, 17, 20, 22–26, 28, 29] and 7 cohort studies [12, 13, 16, 18, 19, 21, 27] (Figure 2). Of the 18 included studies, 8 were conducted in USA [16, 17, 19, 20, 24, 26, 27, 29]; 2 in Australia [15, 22]; 2 in Italy [25, 28]; and 1 in Netherlands, Europe, Denmark, Canada, Sweden, and China [12–14, 18, 21, 23]. A total of 701,857 female subjects, including 8,683 ovarian cancer cases, were included. Most studies matched or adjusted for some potential confounders, including age, education, total energy intake, and use of oral contraceptives (OCPs). The Jadad scores for the included studies ranged from 3–5. Table 1 summarizes the quality scores of the cohort studies and case-control studies.

**Table 1:** Characteristics of the studies included in the meta-analysis

Study	Year	Country	Study period	No. of cases/size	Exposure range	Adjusted RR (95% CI)	Adjustment for covariates	Jadad score
Gosvig CF et al.	2015	Denmark	1995–1999	382/911	≥ 4 cups/day; 0	0.81 (0.48–1.42)	Pregnancy (ever/never), number of pregnancies (linear), oral contraceptive use (ever/never), duration of OCP use (linear)	4
Braem MG et al.	2012	Europe	1992–2000	241/330,849	highest; lowest	1.07 (0.78, 1.46)	OCP, BMI, smoking status, alcohol consumption, total energy intake, duration of breastfeeding, menopausal status, height, and educational level.	4
Nagle CM et al.	2010	Australia	2002–2005	1,271/1,198	≥ 4cups/day; Never	0.71 (0.52–0.97)	Age, education, parity, hormonal contraceptive use, smoking status (current, ex, or non), fruit consumption, vegetable consumption, coffee consumption, consumption of other types of tea	3
Tworoger SS et al.	2008	USA	1976–2004	507/80,253	≥ 2 cups/d; ≤ 1 cup/wk	0.96 (0.70, 1.30)	Age, parity, OCP use, postmenopausal hormone use, tubal ligation, BMI	4
Song YJ et al.	2008	USA	2002–2005	781/1,263	≥1 cup/d; Non	0.92 (0.63–1.33)	Age, county, year of diagnosis/reference date, race/ethnicity, number of full-term pregnancies, duration of hormonal contraception, education, BMI, smoking, tubal ligation/hysterectomy, family history of breast/ovarian cancer	3
Steevens J et al.	2007	Netherlands	1986–2000	280/62,573	≥ 5 cups/d; 1–3 cups/d	0.65 (0.41, 1.03)	Age, use of oral contraceptives (ever/never)	4
Silvera SA et al.	2007	Canada	1980–1985	264/49,613	≥ 4 cups/d; 0 cups/d	1.07 (0.64, 1.79)	Age, smoking history, pack-years of smoking, alcohol intake, education, BMI, parity, participation in vigorous physical activity, menopausal status, OCP use, energy intake, lactose intake, study center, randomization group	3
Baker JA et al..	2007	USA	1982–1998	414/828	≥ 2 cups/day; 0	0.70 (0.51–0.97)	Age, residence, and year of participation	5
Gates MA et al.	2007	USA	1984–2002	577/66,940	> 2/day; ≤ 1/week (servings)	0.63 (0.40, 0.99)	Age, duration of OCP use, parity, history of tubal ligation, smoking status, history of postmenopausal hormone use, physical activity, lactose intake, total energy intake	5
Larsson et al.	2005	Sweden	1987–2004	301/61,057	≥ 2 cups/day; 0 cups/d	0.54 (0.31–0.91)	Age (in months); BMI; education; parity; OCP use; intake of total energy; consumption of fruit, vegetables, milk, liquor, beer, wine, and coffee	5
Jordan et al.	2004	Australia	1990–1993	696/786	≥ 4 cups/day; 0 cups/d	1.10 (0.76–1.61)	Age, age squared, BMI, duration of OCP, parity, smoking, alcohol, education, energy intake.	4
Yen ML et al	2003	Taiwan, China	1993–1998	86/369	Yes; No	0.79 (0.47–1.32)	Age, income during marriage, and education, number of live births was made on the analysis of age at first pregnancy, number of incomplete pregnancies, breastfeeding, OCP use, intrauterine device use	4
Goodman et al.	2003	USA	1993–1999	164/194	≥ 1 cups/week; ≤1 cups/week	0.99 (0.65–1.51)	Age, ethnicity, OCP, tubal ligation	4
Tavani et al.	2001	Italy	1992–1999	1,031/2,411	≥ 1 cups/month; None	0.90 (0.75–1.08)	Study center, year of interview, age, education, parity, age at menopause, OCP, family history of ovarian/breast cancer, BMI, total energy intake	5
Kuper et al.	2000	USA	1992–1997	549/516	≥ weekly; Rarely	1.06 (0.83–1.36)	Age, center activity	4
Zheng et al.	1996	USA	1986–1993	107/35,369	≥ 2 cups/day; Never or monthly	0.98 (0.50–1.90)	Age at menarche, age at menopause, age at first pregnancy, age, education, smoking status, pack-years smoking, physical activity, fruit/vegetable intake, waist/hip ratio, family history of cancer	5
La Vechia et al.	1992	Italy	1983–1990	742/6,147	≥ 1 cups/day; None	1.2 (1.0–1.4)	Age, area of residence, education, smoking, coffee consumption	4
Miller et al.	1987	USA	1976–1983	290/580	≥ 5 cups/day; 0	0.50 (0.2–1.0)	Age, race, religion, smoking, alcohol, OCP use, estrogen use, BMI, age at menarche, age at first pregnancy, parity, age at menopause, type of menopause, years of education, geographical location of hospital, year of interview, no. of lifetime non-obstetric hospital admissions.	3

USA: the United States of America, BMI: body mass index, CI: confidence interval, RR: relative risk, OCP: oral contraceptive pill.

### Main analysis

**Figure 3:**
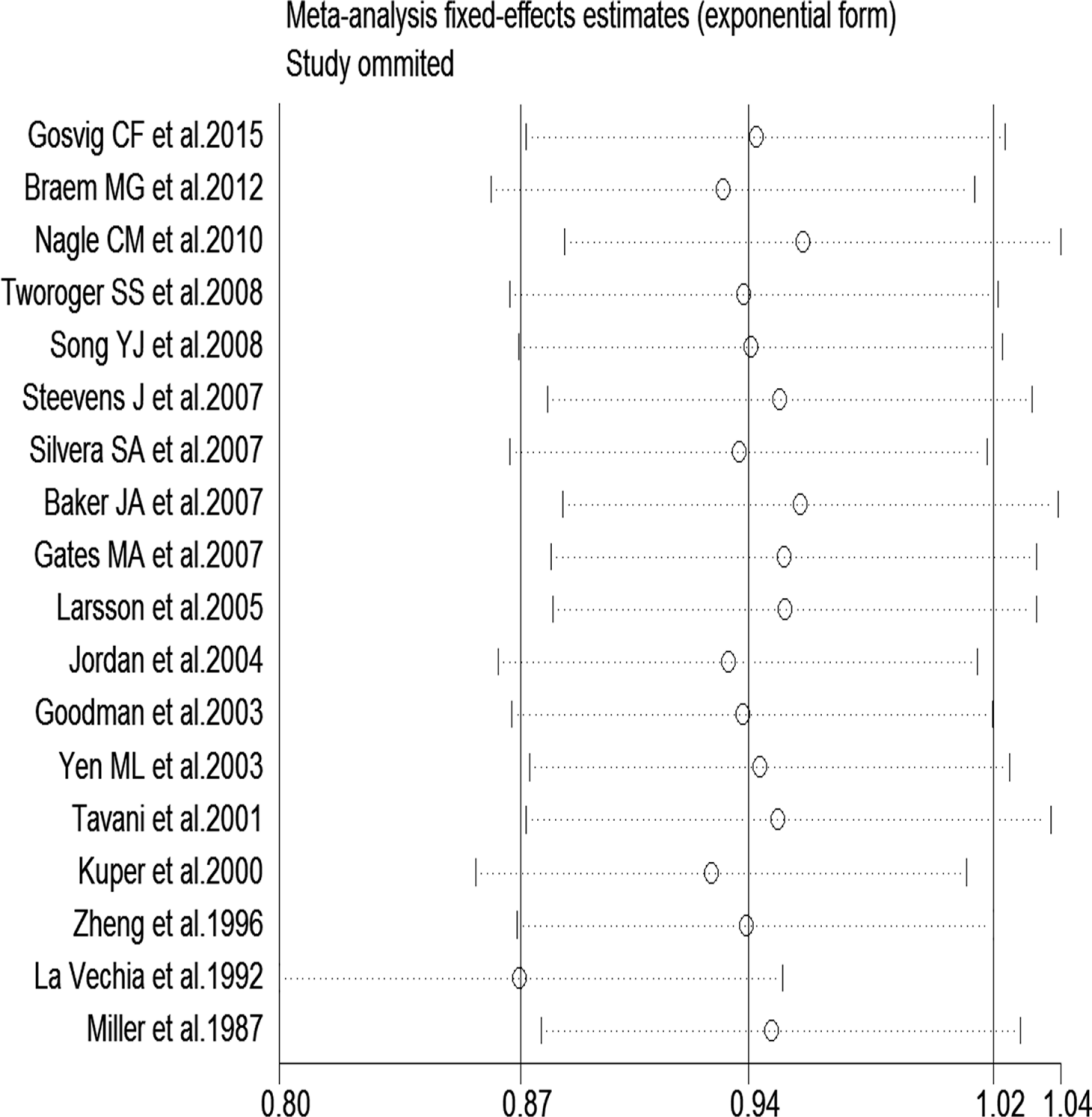
Sensitivity analysis of tea consumption and risk of ovarian cancer showing that omission of any study did not alter the observed effect

In a stratified analysis, we found a statistically significant inverse association between tea consumption and ovarian cancer risk in studies that did not adjust for family history of cancer (RR = 0.85, 95% CI: 0.72, 0.97), education (RR = 0.82, 95% CI: 0.68, 0.96), oral contraceptive (OCP) use (RR = 0.81, 95% CI: 0.71, 0.91), BMI (RR = 0.85, 95% CI: 0.70, 1.00), smoking (RR = 0.83, 95% CI: 0.72, 0.93), and menopause status (RR = 0.85, 95% CI: 0.72, 0.98), in studies with Jadad scores of 3 (RR = 0.76, 95% CI: 0.56, 0.95) and 5 (RR = 0.74, 95% CI: 0.59, 0.89), in cohort study (RR = 0.80, 95% CI: 0.62, 0.97), in Oceania (RR = 0.77, 95% CI: 0.55, 0.99), and in America (RR = 0.84, 95% CI: 0.71, 0.98). Furthermore, statistically significantly inverse associations were identified in studies from the United States (RR = 0.83, 95% CI: 0.69, 0.97) and Sweden (RR = 0.54, 95% CI: 0.31, 0.91) (Table 2). Figure 3 presents the publication dates of the studies. As can be seen from the figure, the results showed that omission of any study could not altered the observed effect, excluding any study, the rest studies’ combined effects still fall within the total combined effect. The results are consistent, and it provides stronger evidence of an effect and of generalizability.

**Table 2:** Stratified analysis of ovarian cancer in relation to tea consumption according to study characteristics

Group	No. of studies	RR (95% CI)	***P***_heterogeneity_	*I^2^* (%)
**Adjustment**				
Family history of cancer				
Yes	3	0.91 (0.76, 1.05)	0.973	0
No	15	0.85 (0.72, 0.97)	0.003	57
Menopause				
Yes	5	0.89 (0.71, 1.07)	0.269	22.8
No	13	0.85 (0.72, 0.98)	0.006	56.5
Education				
Yes	11	0.88 (0.74, 1.03)	0.010	56.9
No	7	0.82 (0.68, 0.96)	0.212	28.4
OCP use				
Yes	14	0.81 (0.71, 0.91)	0.218	21.7
No	4	0.99 (0.72, 1.26)	0.014	71.6
BMI				
Yes	8	0.87 (0.72, 1.02)	0.122	38.6
No	10	0.85 (0.70, 1.00)	0.10	58.2
Smoking				
Yes	8	0.90 (0.70, 1.09)	0.005	65.9
No	10	0.83 (0.72, 0.93)	0.265	19.3
Jadad				
3	4	0.76 (0.56, 0.95)	0.294	19.3
4	9	0.99 (0.86, 1.12)	0.207	26.7
5	5	0.74 (0.59, 0.89)	0.184	35.5
IF				
> 3	9	0.84 (0.70, 0.98)	0.171	30.9
<3	9	0.87 (0.72, 1.03)	0.007	62.2
Study type				
Case-control	11	0.89 (0.76, 1.02)	0.021	52.3
Cohort	7	0.80 (0.62, 0.97)	0.143	37.5
Country				
USA	8	0.83 (0.69, 0.97)	0.178	31.3
Denmark	1	0.81 (0.48, 1.42)	0	0
Australia	2	0.86 (0.49, 1.23)	0.112	60.4
Italy	2	1.04 (0.75, 1.34)	0.023	80.6
Canada	1	1.07 (0.64, 1.79)	0	0
Sweden	1	0.54 (0.31, 0.91)	0	0
Taiwan, China	1	0.79 (0.47, 1.32)	0	0
Netherlands	1	0.65 (0.41, 1.03)	0	0
Geographical region				
Europe	5	0.92 (0.70, 1.15)	0.006	72.1
Oceania	3	0.77 (0.55, 0.99)	0.210	36.0
America	9	0.84 (0.71, 0.98)	0.211	26.2
Asia	1	0.79 (0.47, 1.32)	0	0

IF, impact factor; No., Number; RR, relative risk; CI, confidence interval; USA, the United States of America; BMI, body mass index; OCP, oral contraceptive pill.

### Meta-regression analysis and publication bias

**Figure 4:**
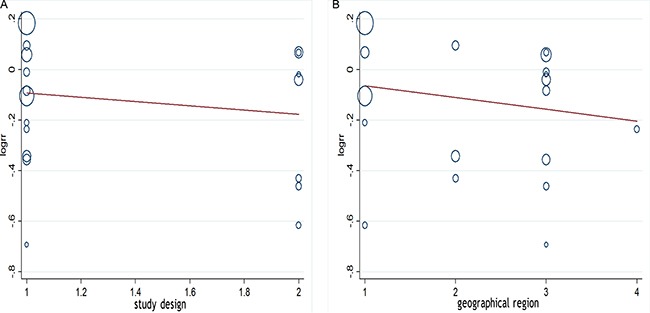
(**A**, **B**)Meta-regulation of study design and risk of ovarian cancer showing that study design was associated with a 47.90% heterogeneity reduction across the studies, and geographical region was associated with a 44.60% heterogeneity reduction across the studies.

**Figure 5:**
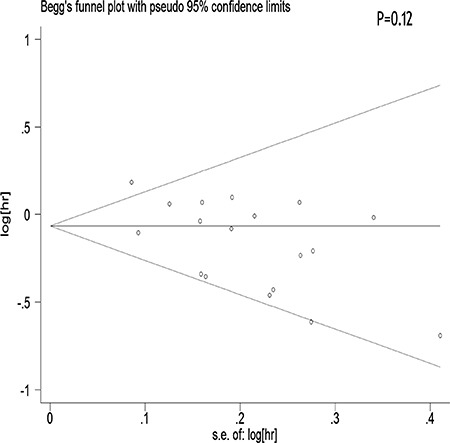
Begger's funnel plot assessing publication bias among the studies

**Figure 6:**
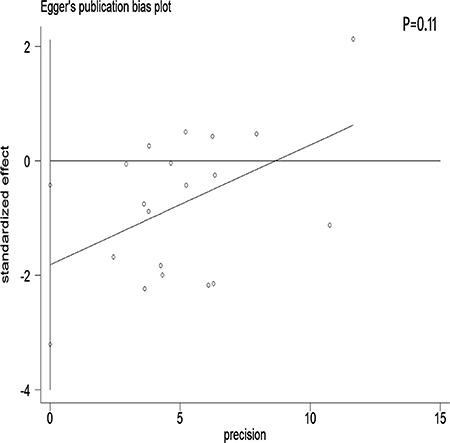
Egger's funnel plot assessing publication bias among the studies

We performed a meta-regression analysis to test the study design and geographic area. We found that study design (47.90%) and geographical region (44.60%) was statistically significant in the multivariate model (Figure 4A, 4B). Interpretation of Figures 5 and 6 revealed no support for publication bias. Furthermore, Begg's and Egger's tests (all *P* > 0.01) indicated no evidence of publication bias among studies (Figures 5 and 6).

## DISCUSSION

This meta-analysis showed that tea consumption significantly reduces the risk of ovarian cancer. Our meta-regression analysis revealed that the study design may be the source of heterogeneity between studies (47.90%), and geographical region was associated with a 44.60% heterogeneity reduction across the studies. In addition, our results were not altered by the control source and most confounder adjustments such as education, OCP use, BMI, and smoking in the subgroup analyses. However, the association was substantially altered by a few confounder adjustments, i.e., family history of cancer, menopause status, and Jadad score.

Stratification by country showed that tea consumption decreased the risk of ovarian cancer only in studies conducted in the United States and Sweden. Geographical differences may be a result of various factors such as the differences in genetic susceptibility, culture, and lifestyles. Moreover, the types of tea and frequency of tea consumption differ among different areas. Our sensitivity analysis indicated that omission of any study did not significantly alter the magnitude of the observed effect, indicating the stability of our findings. Moreover, the Begg's and Egger's tests showed that no publication bias existed.

Many cell *in vitro* experiments and animal experiments have shown that tea has significant anti-cancer effects [30–32]. To a certain extent, a meta-analysis can compensate for the limitations of a single study to resolve the contradiction between the conclusions of the study. Prior meta-analyses also found that tea tends to reduce the incidence of ovarian cancer, but the results were not statistically significant [10–13]; these meta-analyses, selected from the English literature, studied the consumption of different types of tea including green tea and black tea, and the target population was mostly the Caucasian population, which could lead to some bias.

Tea is a relatively inexpensive and safe drink and prevents ovarian cancer by a variety of mechanisms. Tea polyphenols are useful components of the tea extract that can down-regulate the expression of a variety of tumor genes, induce tumor cell apoptosis, block tumor cell cycle, up-regulate the body metabolism, and remove excess free radicals, ultimately playing a role in the prevention and inhibition of tumors [33–36]. Some polyphenols can induce tumor cell apoptosis and inhibit tumor angiogenesis. The consumption of black tea, in particular, which is rich in polyphenols, has been found to significantly reduce the risk of ovarian cancer [37]. However, evidence for the efficacy of black tea for prevention of cancer is not conclusive [12]. On the other hand, green tea contains a variety of phenolic compounds that have a strong antioxidant activity, and its anti-cancer effect is much stronger than that of black tea [13]. One of the main components of green tea is catechol (epigallocatechin gallate), which has remarkable antioxidant activity and can effectively inhibit the growth and evolution of cancer cells [38]. In the Asian population, especially in the Chinese population, the amount of green tea consumed is much higher than that of black tea, but data on this issue are scarce. The effect of tea may also differ due to the difference in genetic heterogeneity and lifestyle among populations. In addition, the various ingredients in tea can have different anticancer activities and effects on different types and subtypes of ovarian cancer [20], which have not yet been explored.

The strength of the present meta-analysis is its large sample size (701,857 female subjects and 8,683 ovarian cancer cases) and no significant evidence of publication bias. Furthermore, our findings were stable and robust. However, there were several limitations that should be noted. First, as a meta-analysis of observational data, the possibility of recall and selection biases cannot be ruled out. Only 5 cohort studies investigated the association between tea intake and ovarian cancer risk, which was a rather small number to draw concrete conclusions from. Compared with case-control studies, cohort studies are less susceptible to bias (e.g., recall bias and selection bias) due to their nature. Therefore, more prospective cohort studies are required on this issue in the future. Second, we did not search for unpublished studies, and only published studies were included in our meta-analysis. Third, publication bias may have occurred, although no publication bias was indicated from both visualization of the funnel plot, the Begg's test and the Egger's test. Fourth, no subanalyses on the histological types of ovarian cancer were performed. Given the heterogeneity of ovarian cancer, this is an important limitation of the study. Fifth, most of the included studies originated from the United States and Europe. Finally, although different types of tea (red, green, and black tea) may have different effects on the risk of ovarian cancer, we did not perform a detailed meta-analysis to determine these differences due to the lack of available studies.

In conclusion, the present meta-analysis of 7 cohort and 11 case-control studies showed that tea consumption was significantly associated with a reduced risk of ovarian cancer. More population-based studies, especially high-quality cohort trials, may be more effective in confirming whether tea consumption prevents ovarian cancer. Further studies in different populations with different tea types in varied dosages are required to correctly ascertain the relationship between tea consumption and the risk of ovarian cancer. Future studies on tea consumption and ovarian cancer risk should focus on the most-common ovarian cancer histotypes separately.

## MATERIALS AND METHODS

### Literature sources and search

A literature search was conducted using MEDICINE, Scopus, PubMed, and Web of Science databases for all relevant studies published in English-language journals up to April 2016. Methodology adhered to the PRISMA guidelines. The search terms included “tea” or “black tea” or “green tea” and “ovary” or “ovarian” and “malignancy” or “neoplasm” or “tumor” or “cancer.” We also reviewed the list of references of each comparative study.

### Study selection

Observational studies that investigated the relationship between tea consumption and ovarian cancer risk were collected independently by two authors. The inclusion criteria were as follows: (i) a case-control or prospective cohort design; (ii) investigation of the association between tea consumption and ovarian cancer incidence; (iii) availability of RR (relative risk) estimates or odds ratios or risk ratios or hazard ratio and 95% CIs. The exclusion criteria were (i) lack of available data and (ii) the following types of articles: news, previews, reports, reviews, comments, and discussions. When there were multiple publications from the same period or in the same population, studies with publication bias were excluded from our meta-analysis. Rate ratio, risk ratio, odds ratio (OR), and hazard ratio (HR) were used as the different measures of RR in all included studies. Because the absolute risk of ovarian cancer is very low, these values were equal to the RR.

### Data extraction and methodological quality assessment

Two authors independently collected the following data from each study: first author of the study, publication date, location of the population studied, study period, number of cases or subjects and study population, exposure range, study-specific adjusted ORs, RRs, or HRs with their 95% CIs for the highest category of tea consumption versus the lowest, confounding factors for matching or adjustments, and Jadad scale from the literature. The methodological quality of the enrolled studies was assessed independently by two authors using the Jadad scale [39].

### Statistical analyses

The RR's adjusted for most confounding factors were used for the association across studies. The heterogeneity was assessed using *I*^2^ statistics. For the I^2^ statistic, heterogeneity was interpreted as absent (I^2^: 0%–25%), low (I^2^: 25.1%–50%), moderate (I^2^: 50.1%–75%), or high (I^2^: 75.1%–100%). Subgroup analyses were performed according to (i) adjustment for family history of cancer (yes/no), adjustment for menopause status (yes/no), adjustment for education (yes/no), adjustment for OCP use (yes/no), adjustment for BMI (yes/no), adjustment for smoking (yes/no); (ii) Jadad scale, impact factor, and study design; and (iii) country where the study was conducted. Pooled RR values and corresponding 95% CIs were estimated using the random-effect model [40], which assumed that the studies included in the meta-analysis had varying effect sizes. To investigate the potential sources of between-study heterogeneity, we carried out a meta-regression analysis and sensitivity analysis by excluding one study at a time to explore whether the results were significantly influenced by a specific study [41]. Publication bias was assessed using Egger's and Begg's tests [42, 43]. However, we did not evaluate a dose-response relationship between tea consumption and ovarian cancer risk due to the lack of date and the difficulty in converting from servings or other units into grams per day. Stata version 13.0 (StataCorp, College Station, TX) was used for statistical analysis. We also performed a meta-regression analysis to test the study design, geographical region.
